# A Pediatric Resident Curriculum for the Use of Health Literacy Communication Tools

**DOI:** 10.3928/24748307-20220517-01

**Published:** 2022-04

**Authors:** Marguerite Costich, Gabriela Bisono, Nicole Meyers, Mariellen Lane, Dodi Meyer, Suzanne Friedman

## Abstract

**Background:**

Despite evidence that use of evidence-based communication tools (EBCT) with a universal precautions approach improves health outcomes, medical trainees report inadequate skills training.

**Objective:**

We developed, implemented, and evaluated a novel, interactive curriculum featuring a 30-minute, single-session didactic with video content, facilitated case-based discussions and preceptor modeling to improve use of EBCT among pediatric residents. A direct observation (DO) skills checklist was developed for preceptors to evaluate resident use of EBCT.

**Methods:**

Shortly after implementation of the curriculum, residents completed a survey assessing self-reported frequency of EBCT use both pre- and post-intervention. DOs were conducted 2 to 3 weeks after the didactic was completed and scores were compared among residents who participated in the curriculum and those who did not. A longitudinal 6-month follow-up survey was also distributed to assess changes over time.

**Key Results:**

Forty-seven of 78 (60%) of residents completed the survey and 45 of 60 (75%) of the eligible residents participated in the DO. There was significant change in self-reported use of all but one EBCT after participation in the curriculum. Residents reported sustained increased frequency of use of all communication tools except for Teach Back, Show Back, and explanation of return precautions in the 6 months following the curriculum. Notably, there was no significant difference in resident scores in the DO among residents who participated in the didactic session and those who did not.

**Conclusions:**

This novel interactive curriculum addresses ACGME (Accreditation Council for Graduate Medical Education) core competencies and fulfills a needed gap in resident curricula for health literacy-related skills training. Findings suggest a small, positive affect on frequency of self-reported use of health literacy EBCT. However, our findings demonstrate a lack of parallel improvement in resident performance during DO. Future curricula may require certain modifications, as well as reinforcement at regular intervals. [***HLRP: Health Literacy Research and Practice*. 2022;6(2):e121–e127.**]

**Plain Language Summary:**

Use of evidence-based communication tools, such as presenting information in small chunks and avoiding complex medical terms among pediatric trainees, is limited. This study describes a new and interactive health literacy curriculum, with emphasis on preceptor modeling and DO to improve use of evidence-based communication tools among residents. After participation in the curriculum, residents report greater use of evidence-based communication tools. However, results from DO of residents did not demonstrate similar improvements.

Nearly 30% of parents have below-basic/basic health literacy, with almost one-half unable to fully perform medication-related tasks (Yin et al., 2009). Due to multiple factors including systemic racism, poverty, and poor access to education and insurance, children of parents with low health literacy have been shown in prior studies to have worse health outcomes including lower glycemic control among patients with diabetes and decreased asthma control among patients with asthma (DeWalt & Hink, 2009; Morrison et al., 2019). The Agency for Healthcare Research and Quality (AHRQ) recommends use of various health literacy-informed verbal and written evidence-based communication tools (EBCT), including Teach Back, Show Back, physical demonstration, use of plain non-medical language, picture and video instructions, and methods such as “chunking,” limiting content, and providing specific action-oriented next-steps to ensure that information is delivered in an organized fashion that is easy to understand (Brega et al., 2015; Morrison et al., 2019). Use of these EBCT has measurable beneficial effects on health outcomes, including improving medication adherence and understanding of diagnoses (Griffey et al., 2015; Yin et al., 2008).

Studies show that while pediatricians are familiar with tools for communication with patients with low health literacy, the extent to which they use them when assessed via direct observation (DO) is limited, owing to barriers such as time limitations and complexity of information (Farrell & Kuruvilla, 2008; Schillinger et al., 2003). Most surveyed pediatricians reported interest in further training, suggesting a need to focus on health literacy training in residency (Turner et al., 2009). Although studies have evaluated implementation of health literacy didactics in other specialty training settings, to our knowledge, no study has assessed pediatric residents' use and training in EBCT, an area of particular importance given the unique triad that exists between parent, patient, and provider. Previously studied curricula have also not explored preceptor modeling, which represents a potentially valuable educational strategy. Additionally, most of these studies did not assess resident skills through DO with patients, making it difficult to draw objective conclusions about changes in practice (Allenbaugh et al., 2019; Coleman, Peterson-Perry, et al., 2017; Green et al., 2014; Kripalani et al., 2006; Pagels et al., 2015)

We describe an interactive health literacy curriculum with emphasis on a universal precautions approach to improve residents' use of EBCT. A DO instrument was created to evaluate curriculum impact on resident utilization of EBCT. The objective of this study was to determine whether curriculum implementation increased resident use of EBCT based on both self-report and a DO exercise. We hypothesized that a targeted curricular intervention would result in an increase in both reported and observed use of EBCT.

## Methods

### Setting and Participants

All 78 pediatric residents have continuity clinic with patient panels and a primary preceptor at one of four New York Presbyterian community-based Ambulatory Care Network practices in Northern Manhattan. There are approximately 20,000 pediatric patients distributed across the four practice sites. The patient population served is predominantly Hispanic and publicly insured. In the community, 21.7% of households have limited English proficiency and 24.6% of households have less than a high school degree (Citizen's Committee for Children of New York, 2018).

### Curriculum Design

Kern's six-step curriculum development approach was used in the creation of the health literacy curriculum (Kern, 2010). A local needs assessment of pediatric residents was conducted in 2018 to assess training in health literacy and self-reported knowledge and utilization of EBCT. Self-reported use of EBCT was limited with only 21% *often*/*always* using Teach Back to assess patient/caregiver understanding. These findings were used to drive curriculum development.

The conceptual framework of social cognitive theory served as the foundation for the curriculum, emphasizing the importance of observation and modeling to enact behavior change (Bandura, 1986). An innovative, interactive curriculum featuring peer-led video examples with associated case-based discussions, preceptor modeling, and a focused DO with provision of immediate feedback was developed for continuity clinic with the aim of creating greater resident self-efficacy using EBCT in future encounters. Curriculum content was advised by the AHRQ Universal Precautions toolkit and consultation with local content experts (Brega et al., 2015).

The first component of the curriculum is an in-person, 30-minute interactive didactic with the following learning objectives: define health literacy, identify risk factors and signs of low health literacy, describe the universal precautions approach, counsel patients using EBCT, and identify health literacy resources for clinical practice. The universal precautions approach of assuming all patients have difficulty understanding health information was emphasized, as literature has shown that most people face some degree of health literacy challenges and that residents are not able to appropriately identify patients with poor literacy skills (Bass et al., 2002; Brega et al., 2015). The didactic features five 2- to 3-minute videos with subsequent discussion questions. Each video discussion focused on specific communication strategies including summarizing information, explaining in small “chunks,” providing task-specific instructions, using plain language, providing pictograms and appropriate written materials, practicing Teach Back and Show Back, and demonstrating certain tasks. These were chosen based on AHRQ recommendations, as well as alignment with previously published health literacy competencies (Brega et al., 2015). Notably, the strategies emphasized in our curriculum have significant overlap but do not entirely match those prioritized by other curricula (Coleman, Hudson, et al., 2017). Our strategies were specifically chosen to address the communication challenges inherent to the pediatrics setting, such as the simultaneous education of parents and children with varying capacity and health literacy. For example, we chose not to include Coleman's strategies related to developing a mutual agenda and eliciting concerns at the start of visits as parents and children may not share the same priorities. We instead emphasized other strategies and skills such as the use of pictograms, visual aids, and appropriate measuring tools, which are particularly relevant to pediatric visits (e.g., how to properly mix formula). Videos were recorded by G.B. using an iPhone and featured other residents and administrative staff at the continuity clinic sites. The video content was reviewed and revised as needed (M.C. and S.F.). These videos were made accessible to all faculty and residents through a dedicated program educational website. The didactic was reviewed by a group of six faculty involved in the development of all clinic educational training materials and revisions were made based on feedback. The didactic launched in October 2019.

The second component of the curriculum consisted of preceptor modeling. Modeling is an effective teaching technique that improves learner skills and competencies and is associated with high rates of resident satisfaction (Huff et al., 2014). Preceptors identified specific scenarios and modeled, either in the examination room or while precepting, how they would review topics with the family using the EBCTs featured in the didactic training. Preceptor modeling was not tracked by individual preceptor, but residents reported if their preceptor used modeling when completing their surveys.

The third component of the curriculum was the creation of a DO tool (**Table A**). It was created, in part, to fulfill a new requirement by the Accreditation Council for Graduate Medical Education (ACGME) for completion of four evaluations in continuity clinics annually. Given the applicability to all patient care interactions and the gap in skills identified by needs assessment, a health literacy focused DO was chosen to meet this need. Skills observed in the DO were directly aligned with selected competencies, including communication skills, patient care, and professionalism, in accordance with best practices in DO (Berz et al., 2017; Hamburger et al., 2011). The specific items included in the DO tool matched the previously outlined learning objectives of our didactic. The DO tool was reviewed by local content experts to establish content validity and modifications were made based on consensus faculty feedback. Interrater reliability among the three faculty preceptors who authored the tool and conducted the faculty development session (M.C., S.F., M.L.) was 0.89 when used to evaluate the video scenarios from the resident didactic, although additional preceptors were not included in this reliability test. Preceptors were asked to observe a portion of a resident's patient encounter and evaluate the resident's use of observed EBCT, with residents not being required to use all tools or strategies listed for any given encounter. The completed DO was later uploaded into MedHub for residents to review.

**Table A:**
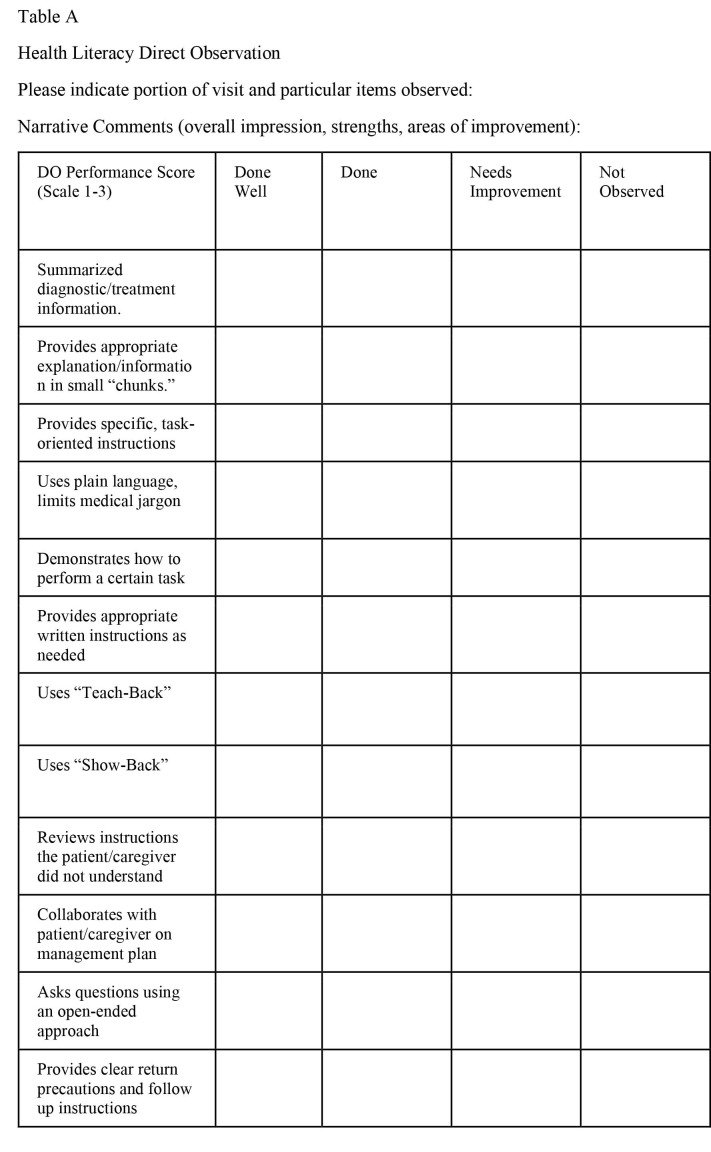
Health Literacy Direct Observation Please indicate portion of visit and particular items observed: Narrative Comments (overall impression, strengths, areas of improvement):

DO Performance Score (Scale 1–3)	Done Well	Done	Needs Improvement	Not Observed
Summarized diagnostic/treatment information.				
Provides appropriate explanation/information in small “chunks.”				
Provides specific, task-oriented instructions				
Uses plain language, limits medical jargon				
Demonstrates how to perform a certain task				
Provides appropriate written instructions as needed				
Uses “Teach-Back”				
Uses “Show-Back”				
Reviews instructions the patient/caregiver did not understand				
Collaborates with patient/caregiver on management plan				
Asks questions using an open-ended approach				
Provides clear return precautions and follow up instructions				

All 18 faculty preceptors attended a required training session related to the curriculum content and its delivery during a faculty division meeting led by M.C., S.F., M.L. Faculty participated in an abridged version of the resident didactic and reviewed all EBCT. Included in the faculty training session was a discussion of preceptor modeling. To help faculty develop criteria for effective evaluation of application of EBCT, the theory of performance-dimension training from workplace-based assessment literature was employed, and the DO was reviewed with all faculty preceptors to orient them to the checklist (Kogan et al., 2015). The DO was also reviewed with all residents during the in-clinic didactic. Faculty were encouraged to share experiences via email including challenges and successes in delivering the curriculum and performing the DO.

Residents participated in the curriculum during a designated week in the context of our residency program's longitudinal continuity clinic curriculum. Modeling by preceptors occurred during that week and the following week. Two to three weeks following the in-clinic didactic, clinic preceptors were asked to complete the DO and provide direct, immediate feedback. Of note, not all residents were present in clinic at the time of the in-person didactic and preceptor modeling because of duty hour restrictions and conflicting clinical responsibilities. However, all residents present in clinic during the designated week participated in the curriculum. The residents who were not in clinic for the didactic constituted the control group for our study.

## Measures

Resident satisfaction with the curriculum and self-reported changes in frequency of use of communication tools were assessed through an anonymous online survey administered to all pediatric residents via Qualtrics (Provo, UT) in the month that followed the in-person didactic (**Table B**). The 5-point Likert scale was used to assess resident satisfaction and self-reported changes. Recent evidence has demonstrated that retrospective pre-post surveys are as effective for program evaluation purposes among residents as traditional pre-post surveys (Bhanji et al., 2012). Only those residents who indicated that they had been present for the in-person didactic were asked survey questions specifically about the curriculum and their self-reported frequency of use of communication tools. A 6-month follow-up survey was also distributed to all residents to assess whether changes in frequency of use of communication tools were sustained. The 6-month follow-up survey was not linked to the retrospective pre-post survey in order to preserve anonymity.

**Table B:**
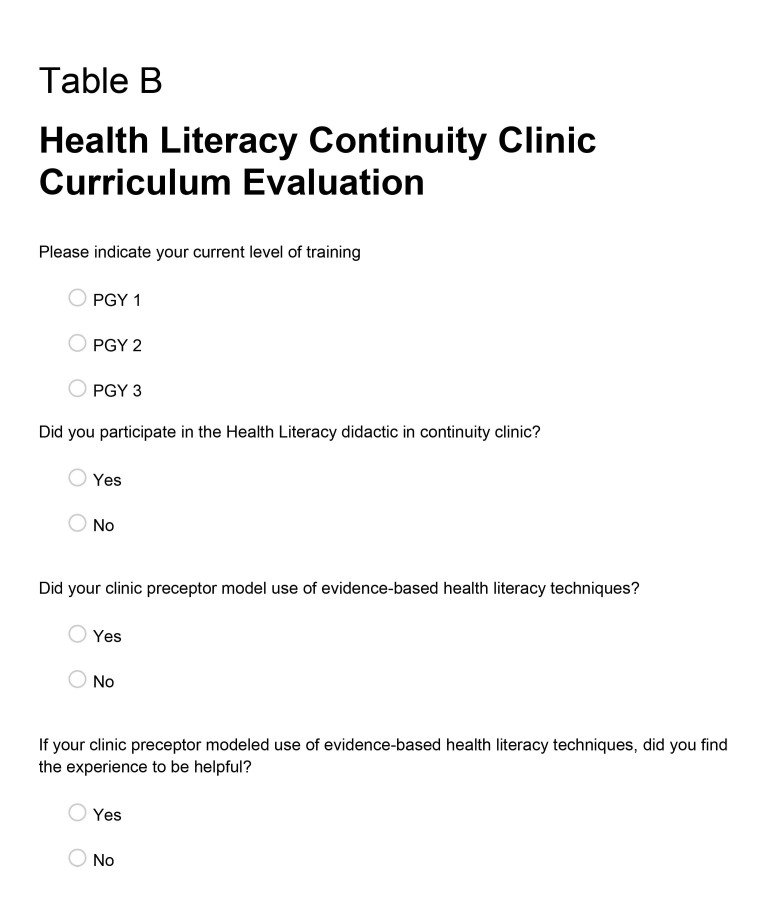

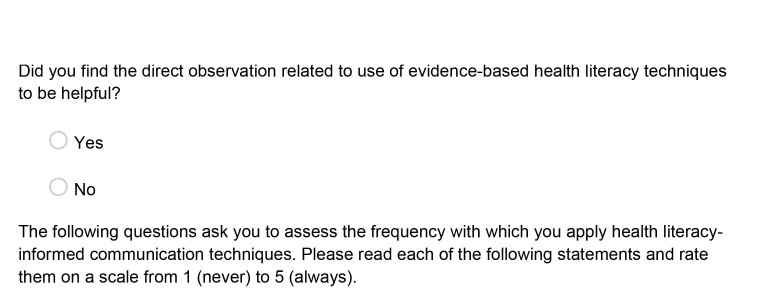

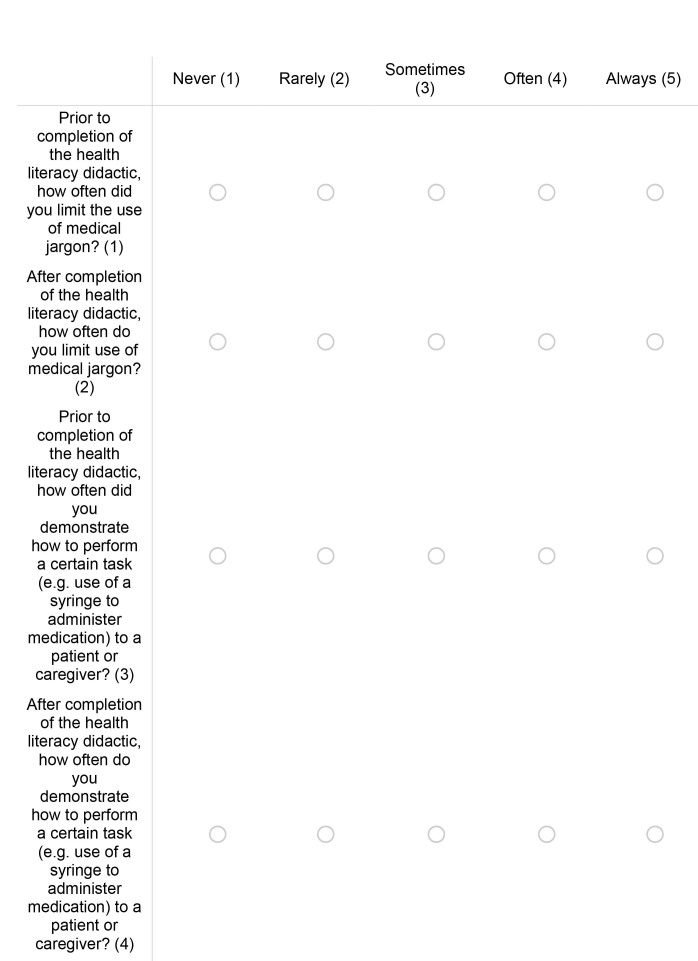

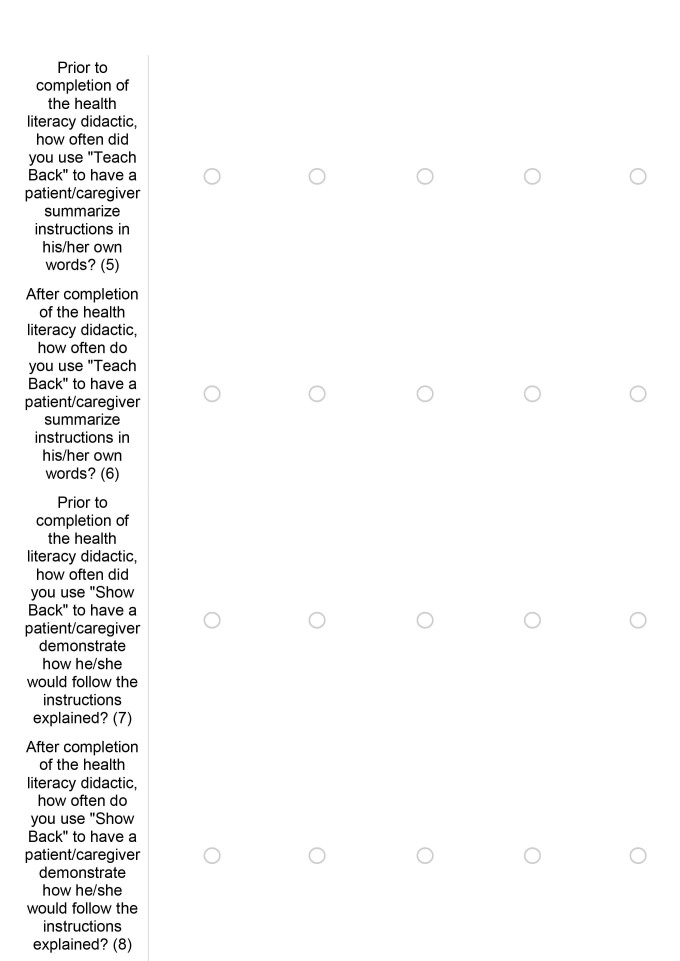

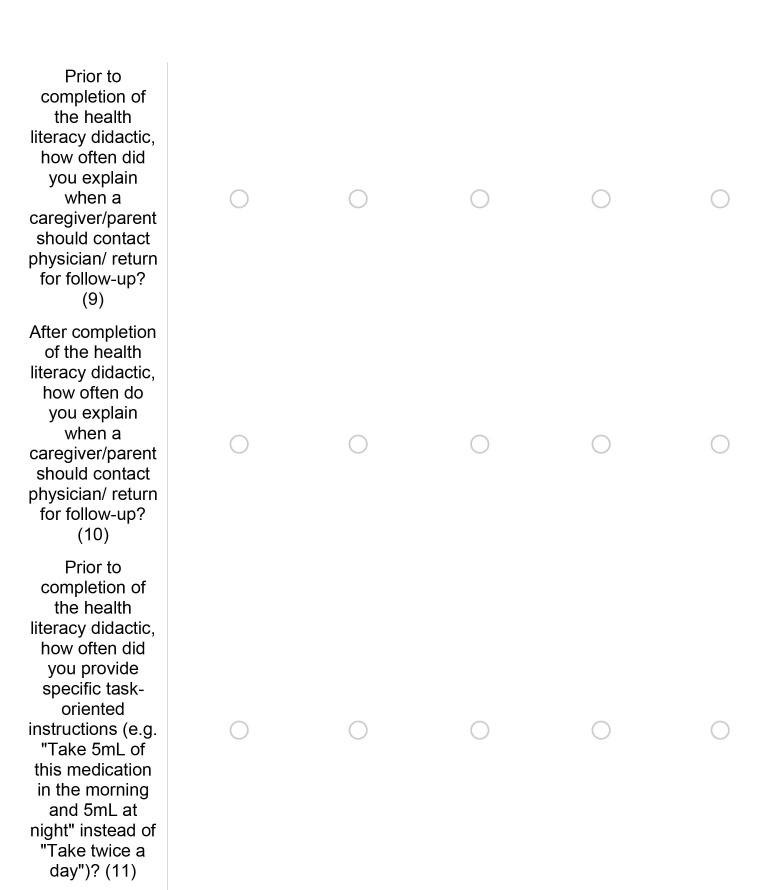

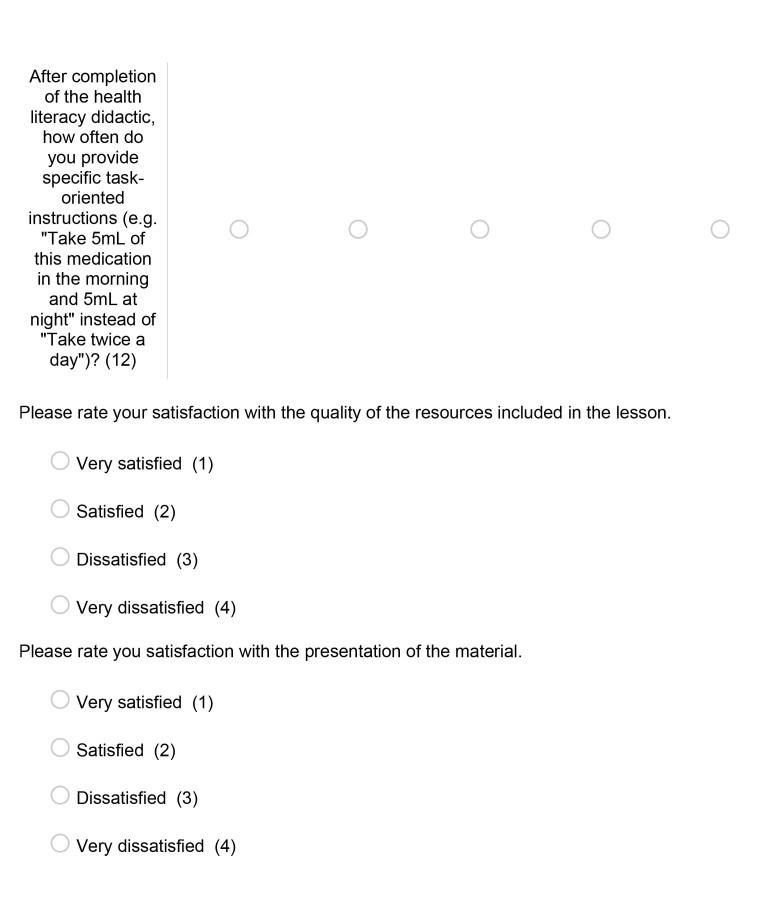

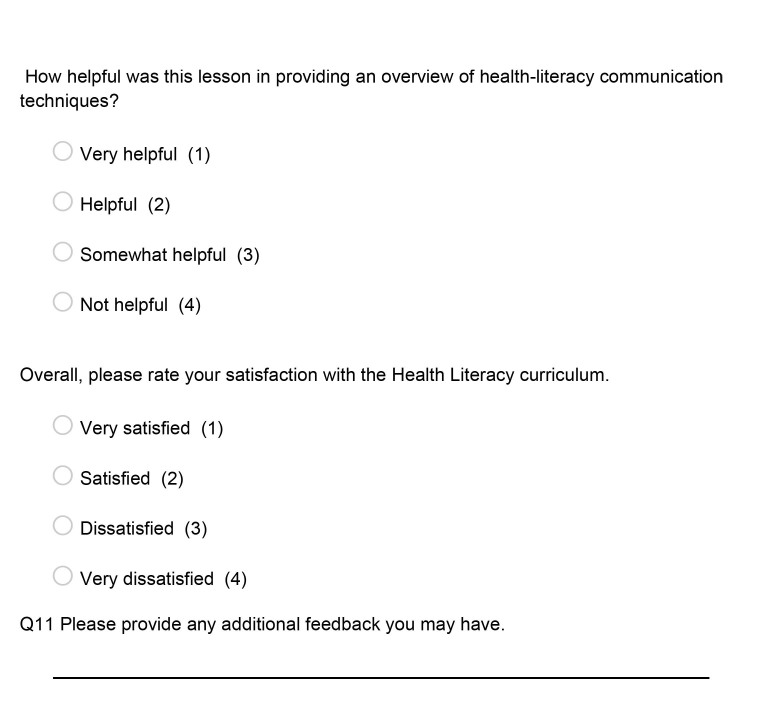
Health Literacy Continuity Clinic Curriculum Evaluation

Please indicate your current level of training 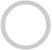 PGY 1 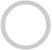 PGY 2 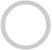 PGY 3
Did you participate in the Health Literacy didactic in continuity clinic? 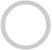 Yes 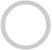 No
Did your clinic preceptor model use of evidence-based health literacy techniques? 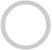 Yes 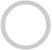 No
If your clinic preceptor modeled use of evidence-based health literacy techniques, did you find the experience to be helpful? 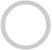 Yes 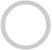 No
Did you find the direct observation related to use of evidence-based health literacy techniques to be helpful? 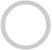 Yes 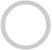 No
The following questions ask you to assess the frequency with which you apply health literacy-informed communication techniques. Please read each of the following statements and rate them on a scale from 1 (never) to 5 (always).

	Never (1)	Rarely (2)	Sometimes (3)	Often (4)	Always (5)
Prior to completion of the health literacy didactic, how often did you limit the use of medical jargon? (1)	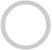	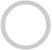	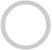	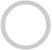	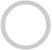
After completion of the health literacy didactic, how often do you limit use of medical jargon? (2)	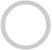	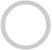	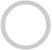	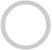	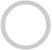
Prior to completion of the health literacy didactic, how often did you demonstrate how to perform a certain task(e.g. use of a syringe to administer medication) to a patient or caregiver? (3)	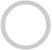	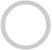	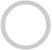	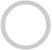	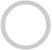
After completion of the health literacy didactic, how often do you demonstrate how to perform a certain task(e.g. use of a syringe to administer medication) to a patient or caregiver? (4)	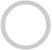	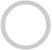	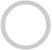	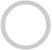	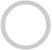
Prior to completion of the health literacy didactic, how often did you use “Teach Back” to have a patient/caregiver summarize instructions in his/her own words? (5)	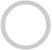	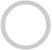	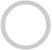	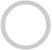	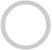
After completion of the health literacy didactic, how often do you use “Teach Back” to have a patient/caregiver summarize instructions in his/her own words? (6)	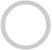	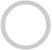	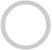	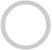	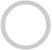
Prior to completion of the health literacy didactic, how often did you use “Show Back” to have a patient/caregiver demonstrate how he/she would follow the instructions explained? (7)	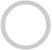	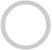	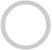	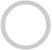	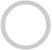
After completion of the health literacy didactic, how often do you use “Show Back” to have a patient/caregiver demonstrate how he/she would follow the instructions explained? (8)	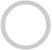	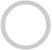	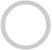	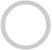	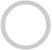
Prior to completion of the health literacy didactic, how often did you explain when a caregiver/parent should contact physician/return for follow-up? (9)	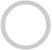	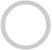	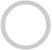	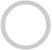	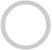
After completion of the health literacy didactic, how often do you explain when a caregiver/parent should contact physician/return for follow-up? (10)	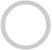	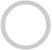	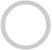	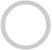	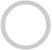
Prior to completion of the health literacy didactic, how often did you provide specific task-oriented instructions (e.g. “Take 5mL of this medication in the morning and 5mL at night” instead of “Take twice a day”)? (11)	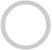	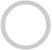	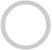	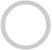	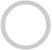
After completion of the health literacy didactic, how often do you provide specific task-oriented instructions (e.g. “Take 5mL of this medication in the morning and 5mL at night” instead of “Take twice a day”)? (12)	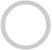	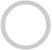	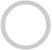	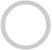	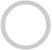

Please rate your satisfaction with the quality of the resources included in the lesson. 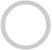 Very satisfied (1) 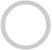 Satisfied (2) 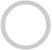 Dissatisfied (3) 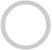 Very dissatisfied (4)
Please rate you satisfaction with the presentation of the material. 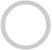 Very satisfied (1) 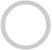 Satisfied (2) 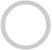 Dissatisfied (3) 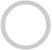 Very dissatisfied (4)
How helpful was this lesson in providing an overview of health-literacy communication techniques? 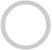 Very helpful (1) 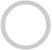 Helpful (2) 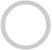 Somewhat helpful (3) 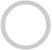 Not helpful (4)
Overall, please rate your satisfaction with the Health Literacy curriculum. 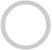 Very satisfied (1) 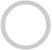 Satisfied (2) 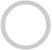 Dissatisfied (3) 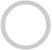 Very dissatisfied (4)
Q11 Please provide any additional feedback you may have. ________________________________________________________________

The DO was administered to all residents, regardless of whether they had been present for the in-clinic didactic. Preceptors completing the DO tool were not blinded to intervention status. Use of the DO was not limited to encounters with English-speaking only patients and could be used for observed encounters in which an interpreter was called. Development and evaluation of the curriculum was approved by the Columbia University Irving Medical Center Institutional Review Board. Residents were consented for participation and use of their data.

### Analysis

The DOs were de-identified by an independent third party prior to analysis, and as such, year of training was not known at time of analysis. The authors of this article excluded their continuity clinic residents from the evaluation. A power analysis was calculated using the findings of the 2018 needs assessment and the assumption that approximately 25% of residents are not present in clinic on their assigned clinic day because of conflicting responsibilities. An effect size of 20% change in the proportion of residents performing a health literacy skill was used in the power calculation. A sample size of 21 curriculum completers and 7 curriculum non-completers was determined to have a power of 0.8 with an alpha of 0.05.

Wilcoxon signed-rank test was used to compare frequency of self-reported use of communication tools and strategies before and after the curriculum. Independent *t*-tests were used to assess for differences in self-reported frequency of use of communication tools prior to the start of the curriculum and at the 6-month follow-up. Mann-Whitney U test was used to compare DO scores among those who did and did not participate in the curriculum as the data was not normally distributed.

## Results

A total of 47 of 78 (60%) of pediatric residents completed the survey, of whom 25% were interns, 34% were second-year residents, and 40% were third-year residents, resulting in a similar distribution across the years of training. Seventy-five percent (*n* = 35) participated in the in-clinic didactic and 79% (*n* = 40) of respondents reported that their clinic preceptors modeled use of communication tools in the clinic. The proportion of first-, second-, and third-year residents who did not participate in the didactic was evenly distributed. Other demographic data were not collected from survey respondents.

There was significant change in self-reported use of all EBCT after participation in the curriculum except for limitation of use of medical jargon (**Table 1**). The 6-month post-survey was completed by a total of 20 of 78 residents, or approximately 25% of residents, with an even distribution between years. Residents continued to self-report significantly increased frequency of use of all communication tools except for Show Back, Teach Back, and explanation of return instructions in the 6 months after the implementation of the curriculum (**Table 1**).

**Table 1:**
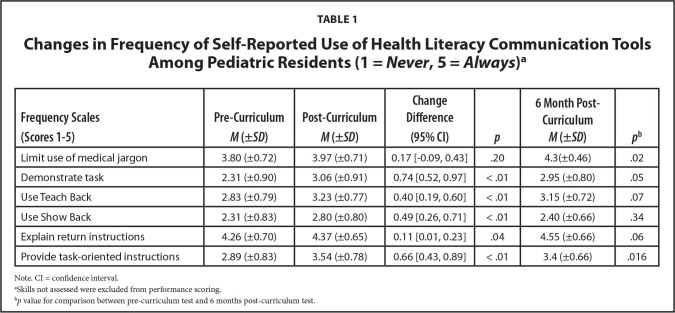
Changes in Frequency of Self-Reported Use of Health Literacy Communication Tools Among Pediatric Residents (1 = *Never*, 5 = *Always*)^a^

**Frequency Scales (Scores 1–5)**	**Pre-Curriculum *M* (±*SD*)**	**Post-Curriculum *M* (±*SD*)**	**Change Difference (95% CI)**	***p***	**6 Month Post-Curriculum *M* (±*SD*)**	***p* ^b^**
Limit use of medical jargon	3.80 (±0.72)	3.97 (±0.71)	0.17 [−0.09, 0.43]	.20	4.3(±0.46)	.02
Demonstrate task	2.31 (±0.90)	3.06 (±0.91)	0.74 [0.52, 0.97]	< .01	2.95 (±0.80)	.05
Use Teach Back	2.83 (±0.79)	3.23 (±0.77)	0.40 [0.19, 0.60]	< .01	3.15 (±0.72)	.07
Use Show Back	2.31 (±0.83)	2.80 (±0.80)	0.49 [0.26, 0.71]	< .01	2.40 (±0.66)	.34
Explain return instructions	4.26 (±0.70)	4.37 (±0.65)	0.11 [0.01, 0.23]	.04	4.55 (±0.66)	.06
Provide task-oriented instructions	2.89 (±0.83)	3.54 (±0.78)	0.66 [0.43, 0.89]	< .01	3.4 (±0.66)	.016

Note. CI = confidence interval.

a Skills not assessed were excluded from performance scoring.

b *p* value for comparison between pre-curriculum test and 6 months post-curriculum test.

Of those residents who participated in the didactic session, 94% were overall *satisfied*/*very satisfied* with the didactic. Mean satisfaction scores for the curriculum, presentation of materials, and resources provided were 1.7 (±0.6), 1.5 (±0.5), 1.5 (±0.5), respectively (1 = *very satisfied*, 4 = *very dissatisfied)*.

A total of 60 DOs were completed at the time of data collection, accounting for 77% of the residents. Forty-five resident observations were included in the analysis, accounting for 75% of total eligible DOs as 15 DOs were not included in the analysis as they were completed by study authors. A majority (*n* = 36, 80%) participated in the didactic session. There was no significant difference in mean scores in the DO among residents who participated in the didactic session and those who did not (**Table 2**).

**Table 2:**
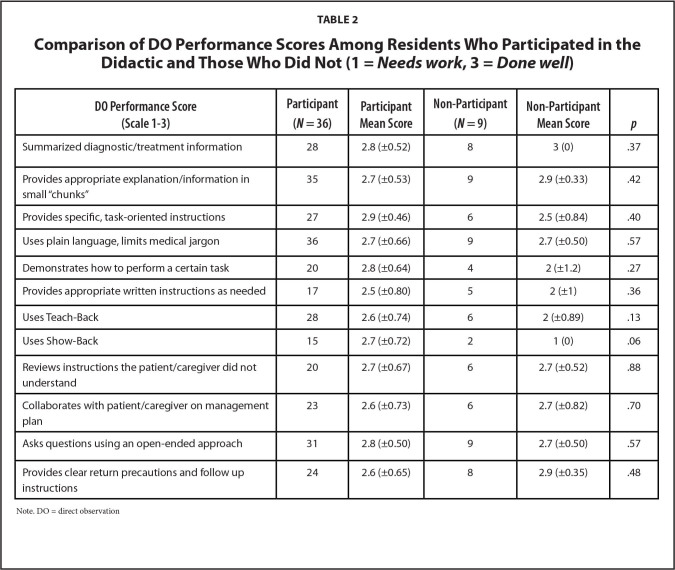
Comparison of DO Performance Scores Among Residents Who Participated in the Didactic and Those Who Did Not (1 = *Needs work*, 3 = *Done well*)

**DO Performance Score (Scale 1–3)**	**Participant (*N* = 36)**	**Participant Mean Score**	**Non-Participant (*N* = 9)**	**Non-Participant Mean Score**	***p***
Summarized diagnostic/treatment information	28	2.8 (±0.52)	8	3 (0)	.37
Provides appropriate explanation/information in small “chunks”	35	2.7 (±0.53)	9	2.9 (±0.33)	.42
Provides specific, task-oriented instructions	27	2.9 (±0.46)	6	2.5 (±0.84)	.40
Uses plain language, limits medical jargon	36	2.7 (±0.66)	9	2.7 (±0.50)	.57
Demonstrates how to perform a certain task	20	2.8 (±0.64)	4	2 (±1.2)	.27
Provides appropriate written instructions as needed	17	2.5 (±0.80)	5	2 (±1)	.36
Uses Teach-Back	28	2.6 (±0.74)	6	2 (±0.89)	.13
Uses Show-Back	15	2.7 (±0.72)	2	1 (0)	.06
Reviews instructions the patient/caregiver did not understand	20	2.7 (±0.67)	6	2.7 (±0.52)	.88
Collaborates with patient/caregiver on management plan	23	2.6 (±0.73)	6	2.7 (±0.82)	.70
Asks questions using an open-ended approach	31	2.8 (±0.50)	9	2.7 (±0.50)	.57
Provides clear return precautions and follow up instructions	24	2.6 (±0.65)	8	2.9 (±0.35)	.48

Note. DO = direct observation

## Discussion

We developed a novel, interactive curriculum on health literacy focused EBCT that addresses ACGME competencies. We assessed pediatric residents' use of health literacy EBCT after implementation of the curriculum through self-evaluations and DO. The curriculum addresses a clear need as most health literacy curricula are not focused on pediatric specific skills, do not incorporate preceptor modeling, and do not include an assessment of skill use with patients, but rather use of standardized patient encounters and role-playing (Allenbaugh et al., 2019; Coleman, Peterson-Perry, et al., 2017; Green et al., 2014; Howley et al., 2008; Kripalani et al., 2006; Pagels et al., 2015).

We have demonstrated that the in-person didactic portion of the curriculum was well-received and that resident's self-reported frequency of use of EBCT increased after its implementation. However, sustained use of all EBCT, particularly of Teach Back and Show Back was not achieved, perhaps reflecting previously identified challenges of incorporating communication tools into practice. Prior studies have also demonstrated limited long-term effects (Coleman, Peterson-Perry, et al., 2017)

We developed a brief, health literacy-focused DO checklist both to fulfill the ACGME requirements for an additional DO in resident continuity clinic and serve as an evaluative tool for the health literacy curriculum. Literature has demonstrated that DOs play a critical role in formative feedback, and we are hopeful that the DO spurred discussion of use of EBCT among residents (Donato, 2014). This DO, with its focus on use of high-value, EBCT, can also be flexibly used and implemented by other programs and in other settings.

There was no difference in directly observed skills for residents who participated in the didactic and those that did not. It is possible that residents may be using EBCT more often than realized, or alternatively, that residents are overestimating their skills using EBCT. This corroborates the literature that physicians are not reliable at assessing their own skills and underscores the need for objective assessments (Davis et al., 2006). This also suggests our didactic may require modifications to ensure observable changes in resident EBCT. It is conceivable that modeling of skills by preceptors was the primary driver of skill change, accounting for strong performances by all residents on the DO. All residents, even those not present for the in-person didactic, likely benefited from preceptor modeling throughout their continuity clinic experience, as evidenced by a greater number of residents reporting preceptor modeling than presence during the didactic portion curriculum. Lastly, the Hawthorne effect must be recognized as a potential contributor to residents' overall strong performance on the DO.

This study has several limitations worth noting. The curriculum was implemented at a single institution, potentially limiting generalizability. Although DOs were not limited to encounters with English-speaking patients, we do not have data on language or interpreter use, which could influence the use of certain EBCT. Additionally, the short-term improvement in self-reported use of EBCT is promising but may reflect the effects of social-desirability and/or response shift bias, as the study was appropriately powered. Further, the response rate of the 6-month follow-up survey was quite low and differed quite substantially from that administered immediately after implementation of the curriculum. The follow-up survey was administered in the spring, around the height of the coronavirus disease 2019 pandemic in New York City at a time when residents had been pulled from outpatient clinic to meet health system demands. It is possible that competing demands influenced resident participation.

The DO tool was created locally by our team and lacks evidence of validity. Total number of DOs was also lower than we had hoped, with only 75% of eligible residents having DOs in MedHub. While we had hoped that this brief, focused DO would provide a sustainable approach for assessing residents in the ambulatory setting, it is clear there needs to be greater faculty development to improve completion rates of DOs. Despite these limitations, the aim of this project was to create a novel health literacy curriculum and this goal was achieved.

Future work should focus on validating the use of the DO as well as addressing challenges in implementation of the curriculum. A longitudinal curriculum with reinforcement of health literacy focused EBCTs at regular intervals should be considered. The modeling component of the curriculum would also benefit from more careful tracking, as well as a standardized guide that preceptors could follow. Lastly, the videos used in our study can certainly be used in other pediatric outpatient clinics; however, if used in other settings, modifications should be made.

## Conclusions

Through the development of a novel, interactive health literacy curriculum based on ACGME competencies and preceptor modeling, we were able to improve residents' self-reported skills. However, our results from DO of residents did not match their self-reported improvements, highlighting the possibility that self-reported health literacy skills are unreliable. Given our curriculum did not result in significant observed behavior changes, future curricula may require additional modeling, reinforcement, and incentivization to achieve success.
